# PCSK9 Regulation of Lipid Metabolism in the Nervous System: Implications for Schwann Cell Function and Peripheral Neuropathy

**DOI:** 10.3390/cells14181479

**Published:** 2025-09-22

**Authors:** Agnieszka Nowacka, Maciej Śniegocki, Ewa A. Ziółkowska

**Affiliations:** 1 Department of Neurosurgery, Collegium Medicum in Bydgoszcz, Nicolas Copernicus University in Toruń, ul. Curie Skłodowskiej 9, 85-094 Bydgoszcz, Poland; sniegocki@cm.umk.pl; 2 Department of Pediatrics, School of Medicine, Washington University in St. Louis, St. Louis, MO 63110, USA

**Keywords:** PCSK9, Schwann cells, lipid metabolism, CD36, peripheral neuropathy, nervous system, myelination

## Abstract

Neural function relies on tightly regulated lipid metabolism to sustain membrane integrity, synaptic signaling, and energy production. Myelinating glia, particularly Schwann cells, require continuous lipid flux to build and maintain myelin, rendering them vulnerable to imbalances between lipid entry and oxidative capacity. Proprotein convertase subtilisin/kexin type 9 (PCSK9), widely studied in hepatic cholesterol regulation, has emerging roles in the nervous system. In the central nervous system (CNS), local PCSK9 expression influences low-density lipoprotein receptor (LDLR) family abundance, neuronal survival pathways, and neuroinflammatory tone, although circulating PCSK9 has limited parenchymal access due to the blood–brain barrier (BBB). In the peripheral nervous system (PNS), recent evidence highlights a PCSK9–CD36 axis in Schwann cells; genetic Pcsk9 loss elevates CD36, increases fatty-acid influx, promotes lipid droplet expansion and acylcarnitine accumulation, and triggers mitochondrial stress that manifests as hypomyelination, C-fiber pathology, and selective small-fiber neuropathy. These findings suggest that PCSK9 normally restrains CD36-dependent transport to align lipid supply with metabolic demand. Clinically, PCSK9 inhibitors have demonstrated cardiovascular benefit without major neurocognitive signals, yet small-fiber outcomes have not been systematically assessed. This review integrates current evidence on PCSK9 biology across neural compartments, highlights mechanistic links to Schwann cell lipid handling, and outlines research priorities to resolve neural safety and therapeutic potential in lipid-driven neuropathies.

## 1. Introduction

Neural function depends on lipids that build membranes, support signaling, and fuel metabolism [1,2,3,4]. The brain is the most cholesterol-rich organ, and myelin that encloses axons in the central and peripheral nervous systems has an unusually high lipid-to-protein ratio, which promotes membrane compaction, fast impulse conduction, and metabolic stability [5]. Because lipid synthesis, transport, and degradation must be precisely coordinated, even modest shifts in these pathways can alter membrane composition and energy balance and can impair myelination and synaptic function, which in turn may contribute to neurodevelopmental abnormalities or progressive neurodegeneration [1,6,7,8]. In the peripheral nervous system (PNS), Schwann cells are especially sensitive to changes in lipid supply and utilization, since they assemble large myelin segments while maintaining mitochondrial function and redox homeostasis, so stable lipid balance is a central determinant of neural health across both compartments [8,9,10,11].

Proprotein convertase subtilisin/kexin type 9 (PCSK9) is best known for its hepatic action that promotes low-density lipoprotein receptor (LDLR) degradation and shapes plasma LDL cholesterol, which explains the clinical success of PCSK9 inhibitors in cardiovascular medicine [8,12,13,14,15]. A growing body of work indicates that PCSK9 is also relevant in neural tissues. In the central nervous system (CNS), its expression is developmentally regulated, remains detectable in selected adult regions and in the cerebrospinal fluid, and has been linked to neuronal differentiation, inflammatory signaling, and local lipid handling through LDL receptor family members such as ApoER2 and VLDLR [3,4,8,16,17,18]. In the peripheral nervous system, transcriptional and proteomic studies indicate the meaningful expression of PCSK9 in Schwann cells and in human peripheral nerves. Mouse models with constitutive PCSK9 loss show reduced thermal and mechanical sensation, the hypomyelination of small fibers, axonal abnormalities within Remak bundles, and fewer nociceptive Schwann cells [8]. These changes occur together with higher CD36 abundance, accumulation of neutral lipids, altered mitochondrial ultrastructure, and buildup of acylcarnitines in peripheral nerves. Although only a few individuals with complete PCSK9 loss have been described, and detailed sensory testing remains limited, the preclinical evidence supports a role for PCSK9 in neural lipid homeostasis with Schwann cells as a likely focal point [3,15,19,20,21,22,23].

This review integrates PCSK9 biology with the lipid demands of neural tissues and evaluates how PCSK9 may shape lipid metabolism that affects myelination and peripheral nerve integrity. We highlight a knowledge gap around a putative PCSK9–CD36–mitochondrial-stress axis in Schwann cells. We first summarize the PCSK9 structure, maturation and regulation, and receptor routing principles that may extend beyond the liver to neural receptors. We then map neural lipid handling by contrasting CNS and PNS constraints, covering LDL-receptor family and ApoE pathways, CD36-mediated fatty-acid uptake, ABCA1/ABCG1-mediated cholesterol efflux, and the mitochondrial needs of myelinating glia. Building on this, we review PCSK9 in the CNS and focus on the PNS, where evidence points to dysregulated Schwann-cell lipid flux and a mechanism linking PCSK9 to CD36-dependent lipid entry and mitochondrial overload that may drive hypomyelination and small-fiber injury. Finally, we discuss the clinical implications for PCSK9 inhibitors, differences between pharmacologic inhibition and genetic deficiency, and experimental and translational priorities to clarify neural safety and enable targeted control of lipid flux.

While previous reviews have primarily discussed PCSK9 in the context of hepatic lipid metabolism or central nervous system regulation, few have addressed its potential roles in the peripheral nervous system. To our knowledge, no prior review has focused specifically on Schwann cells and their vulnerability to lipid imbalance. By centering on this glial population and its link to CD36-dependent lipid entry, our article highlights a novel perspective that distinguishes it from earlier work and aims to clarify how PCSK9 may contribute to peripheral neuropathy.

## 2. PCSK9 Biology: Structure, Regulation, and Canonical Functions

### 2.1. Domain Architecture and Activation of PCSK9

PCSK9 is a secreted subtilisin-like protein built from a signal peptide, an N-terminal prodomain, a catalytic domain, and a C-terminal Cys-His-rich domain (CHRD) composed of three modules known as M1, M2, and M3 [24,25,26]. In the endoplasmic reticulum, the zymogen undergoes an autocatalytic cut at the prodomain–catalytic junction, the cleaved prodomain remains tightly bound and shields the active site, so the secreted protein acts mainly through binding to receptors rather than by proteolysis. This architecture underlies the paradox of a “protease” that regulates receptors via escorting and routing rather than enzymatic cleavage. The catalytic domain contacts the LDLR at its EGF-A repeat, and the C-terminal CHRD contributes to receptor engagement and trafficking [27]. A second layer of maturation occurs through extracellular processing by furin. Furin cleavage generates a shorter circulating species that, depending on the context, shows reduced affinity for LDLR and lower activity, although several studies indicate that cleaved PCSK9 can still direct LDLR degradation in vitro and in vivo [28,29,30,31]. Both intact and furin-cleaved forms circulate in humans, which has implications for activity readouts and assay interpretation [30,31,32,33,34].

### 2.2. Transcriptional Control by SREBP2 and HNF1α, and Post-Transcriptional Regulation

PCSK9 transcription is linked to cellular sterol status through sterol regulatory element-binding protein 2 (SREBP2), which binds a sterol-responsive element in the PCSK9 promoter and raises expression when intracellular cholesterol is low. Hepatic hepatocyte nuclear factor 1α (HNF1α) cooperates with SREBP2 to amplify PCSK9 transcription, which helps explain why statins increase PCSK9 more strongly than LDLR in some settings. This feedback is a central reason why PCSK9 inhibitors combine well with statins in the clinic [35,36,37,38,39]. Outside the liver, regulation is more diverse. During neural differentiation, PCSK9 mRNA can rise without a parallel increase in LDLR protein, which points to alternative transcriptional programs and to post-transcriptional control in neural cells. MicroRNAs add another layer: several miRNAs directly target the PCSK9 3′-untranslated region (3′-UTR) and reduce its expression in cells and animal models, including miR-483-5p and a set of others reported in high-throughput screens. Together, these controls help explain why PCSK9 and LDLR levels are sometimes uncoupled in extrahepatic tissues [3,26,37,40,41,42].

### 2.3. Secretion, Circulating Forms, and Tissue Distribution

**Figure 1:**
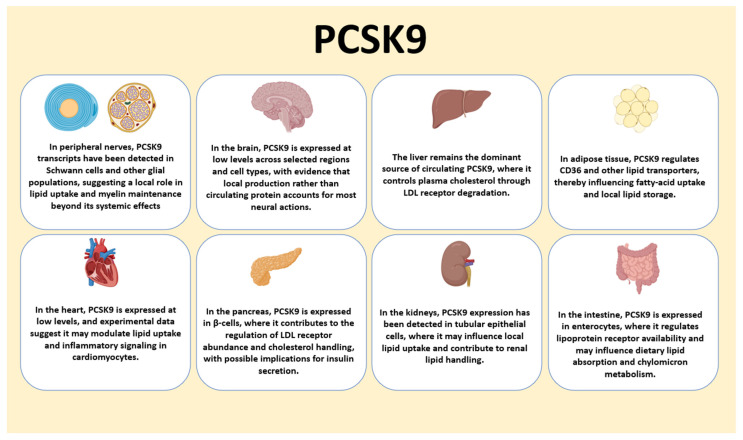
Extrahepatic expression and functions of PCSK9 across neural and metabolic tissues.

The liver is the dominant source of circulating PCSK9, acting as an endocrine hub that releases the protein into plasma, while extrahepatic expression is detectable in intestine, kidney, pancreas, adipose, and vascular cells, and in selected neural populations [3,4,8] (Figure 1). These extrahepatic pools likely support paracrine or autocrine control of receptor routing in local microenvironments and help explain tissue-specific phenotypes that cannot be accounted for by hepatic output alone [4,8,43,44,45,46].

In the circulation, PCSK9 exists as intact full-length and furin-cleaved species and distributes between a free fraction and a lipoprotein-bound fraction. Binding to apolipoprotein B (apoB)-containing particles, most prominently LDL and, in many individuals, lipoprotein(a), appears to stabilize PCSK9 and may influence its residence time and targeting to hepatocyte LDL receptors [21,31,46,47,48]. This partitioning is not fixed, it varies with metabolic state and with therapies that alter lipoprotein profiles, which is relevant when interpreting pharmacodynamic readouts or comparing assays that capture free versus total PCSK9 [30,36,49].

Secretion follows the classical secretory pathway, newly synthesized PCSK9 undergoes intramolecular autocatalytic processing in the endoplasmic reticulum, the prodomain remains non-covalently bound and chaperones the catalytic domain, and the complex is then trafficked through the Golgi and released. Once extracellular, PCSK9 acts primarily through protein–protein interactions rather than proteolysis, engaging surface receptors and diverting them from recycling to lysosomal degradation. Systemic PCSK9 levels vary with physiological cues, such as sterol-responsive transcription and commonly used lipid-lowering drugs, and these shifts further alter the balance between free and particle-bound pools [25,36,37,49,50,51].

Tissue distribution introduces an important nuance for the nervous system; although plasma PCSK9 orchestrates whole-body lipoprotein handling, parenchymal effects in the brain and peripheral nerves are expected to arise mainly from locally produced protein because access from the circulation is limited [3,15,25,52].

When it comes to the summary of PCSK9 expression beyond the liver, in peripheral nerves, PCSK9 transcripts are detected in Schwann cells and other glia, suggesting roles in lipid uptake and myelin maintenance. In the brain, local PCSK9 expression is low but may regulate neuronal survival and receptor availability. The liver remains the dominant source of circulating PCSK9 through control of LDL receptor degradation. In adipose tissue, PCSK9 influences CD36 and lipid storage. In the heart, low-level expression may modulate lipid uptake and inflammatory signaling in cardiomyocytes. In the pancreas, PCSK9 in β-cells regulates LDL receptor abundance and cholesterol handling with possible effects on insulin secretion. In the kidneys, expression in tubular epithelial cells may affect local lipid handling. In the intestine, PCSK9 expressed in enterocytes regulates lipoprotein receptors and may shape dietary lipid absorption and chylomicron metabolism.

### 2.4. Canonical Receptor Biology: LDLR Turnover and Systemic Cholesterol Control

PCSK9 binds the EGF-A repeat of LDLR at the cell surface and stabilizes the complex in endosomes, which prevents LDLR recycling and diverts the receptor to lysosomes for degradation. This routing does not require PCSK9 protease activity, because catalytically inactive variants still promote LDLR loss, and it is favored by the acidic pH of endosomes that strengthens PCSK9–LDLR binding [50,51,53]. Besides the extracellular route, an intracellular pathway has been described in which PCSK9 encounters LDLR in the secretory pathway and directs it to degradation before reaching the surface. Together, these mechanisms reduce cell-surface LDLR and lower hepatic LDL uptake, which raises plasma LDL cholesterol, conversely, PCSK9 inhibition increases LDLR and lowers LDL cholesterol [26,53,54]. Recent work adds detail to endosomal sorting. PCSK9 binding can interfere with recycling adaptors, such as sorting nexin 17 (SNX17), that normally return LDLR to the membrane, thereby biasing the receptor toward lysosomal delivery. These insights align with the dominant physiological role of hepatic PCSK9 in setting systemic LDL levels [37,51].

### 2.5. Non-Hepatic Targets and Interactors: VLDLR, LRP1, ApoER2, CD36

The routing logic that PCSK9 applies to LDLR extends to other receptors. In cells that express very low-density lipoprotein receptor (VLDLR) and apolipoprotein E receptor 2 (ApoER2), PCSK9 lowers their abundance and reduces ligand signaling, an effect seen in neuronal contexts and consistent with shared structural features across the LDLR family [26,55]. Evidence also supports the PCSK9-mediated downregulation of low-density lipoprotein receptor-related protein 1 (LRP1), a multifunctional endocytic receptor with roles in lipid handling and signaling [56]. The common principle is that PCSK9 binding biases these receptors toward lysosomal degradation after endocytosis [37,55,57]. PCSK9 also engages CD36, a scavenger receptor that mediates long-chain fatty-acid uptake and lipid scavenging. In hepatocytes and adipocytes, PCSK9 promotes CD36 degradation through lysosomal and proteasomal pathways, whereas Pcsk9-deficient mice show increased CD36 and enhanced fatty-acid uptake with greater triglyceride storage [58]. This regulatory link is mechanistically distinct from LDLR control but follows the same routing logic—more PCSK9 means fewer receptors at the surface and lower lipid influx, and less PCSK9 means the opposite. In tissues that depend on balanced lipid entry and oxidative capacity, such as myelinating glia, shifts in CD36 abundance could therefore have outsized metabolic and structural consequences [3,8,20,58]. Endogenous modulators further tune PCSK9 action. Annexin A2 binds PCSK9 via the C-terminal region and limits PCSK9-enhanced LDLR degradation, and its overexpression raises LDLR levels in vivo. Such interactors illustrate how accessory proteins, endocytic adaptors, and the local lysosomal environment together set the fate of receptors that encounter PCSK9 in a given tissue [59,60].

## 3. Lipid Homeostasis in the Nervous System

### 3.1. Lipid Requirements of Neurons and Glia: Cholesterol, Sphingolipids, Plasmalogens

Neural cells depend on a distinct lipid economy that supports membrane architecture, signal transduction, and energy balance. Myelin membranes produced by oligodendrocytes in the CNS and Schwann cells in the PNS are especially lipid rich and consistently approximate a 40:40:20 molar ratio of cholesterol, phospholipids, and glycolipids, unlike most biological membranes that average about 25:65:10, which underscores the extraordinary demand for sterols and sphingolipids in compact myelin [11,61,62,63]. Cholesterol is the single most abundant lipid in myelin, and its enrichment supports membrane compaction and saltatory conduction, while complex sphingolipids and ethanolamine plasmalogens stabilize the multilamellar sheath and modulate redox sensitivity [62]. Neurons require steady access to cholesterol for synaptogenesis and axonal growth but produce less than astrocytes, which function as net sterol suppliers through local lipoprotein systems that move cholesterol between cell types [5,18,64,65]. When lipid flows are perturbed, conduction slows, synapses weaken, and repair programs fail, as summarized in recent reviews that connect myelin lipid dysmetabolism with leukodystrophies, neuropathies, and acquired neurodegenerative states [11,62,66,67].

Peroxisomes contribute essential building blocks to neural membranes by handling very-long-chain fatty acids and plasmalogen synthesis, and inherited peroxisomal disorders highlight how vulnerable the brain and nerves are to defects in these pathways [68]. In parallel, microglia and astrocytes fine-tune lipid storage and export during surveillance and injury, so local changes in lipid composition can alter inflammatory tone and neuronal excitability [62,69,70,71,72].

### 3.2. Uptake and Synthesis in the CNS and PNS Under Barrier Constraints

The brain largely relies on in situ cholesterol synthesis because circulating lipoproteins do not cross the blood–brain barrier efficiently, so astrocytes synthesize cholesterol and package it into apolipoprotein E–containing particles that neurons internalize via LDL receptor family members [64]. Long-chain fatty acids can enter the CNS, yet transport is tightly regulated by endothelial systems that include protein carriers such as major facilitator superfamily domain-containing protein 2A (MFSD2A) for lysophosphatidylcholine-DHA and auxiliary fatty acid–binding proteins (FABPs) and fatty acid transport protein (FATP) pathways [73,74]. The blood–nerve barrier (BNB) and perineurial barrier in peripheral nerves are architecturally distinct from the BBB and display different permeability and transporter profiles, which shapes how circulating lipids and drugs reach endoneurial compartments [75,76,77]. Comparative barrier work places the BNB as highly restrictive yet heterogeneous across sites like dorsal root ganglia, and catalogs tight-junction repertoires that include claudins and ZO proteins relevant for nerve homeostasis [67,68]. These anatomical and transport differences mean that receptor availability and local synthesis have different leverage in the CNS versus the PNS, with Schwann cells combining de novo synthesis and receptor-mediated uptake to sustain the growth and maintenance of myelin [62,69,78,79].

### 3.3. Key Transporters and Receptors in Neural Lipid Traffic

#### 3.3.1. LDLR Family: LDLR, VLDLR, LRP1, ApoER2

LDLR and related receptors orchestrate lipoprotein uptake and signaling in neural tissue. Apolipoprotein E receptor 2 (ApoER2) and VLDLR mediate reelin signaling that guides neuronal migration and supports adult synaptic plasticity, while also serving as lipoprotein receptors that move cholesterol and phospholipids to neurons and glia [44,45,80,81]. Low-density lipoprotein receptor-related protein 1 (LRP1) integrates lipid handling with proteostasis and vascular functions and participates in amyloid-β trafficking and clearance, which links receptor availability to homeostatic and disease processes [80,82,83,84]. Because PCSK9 can lower the surface levels of LDLR-family receptors, local PCSK9 expression has the potential to reshape lipid flux and receptor-dependent signaling in neural cells [3,37,85].

#### 3.3.2. ApoE-Containing Lipoproteins in the Brain

Astrocytes assemble ApoE-rich lipoprotein particles that distribute cholesterol and phospholipids to neurons, which supports synapse formation, axonal growth, and ongoing membrane repair [64]. These CNS lipoproteins differ from plasma particles because they are produced locally and engage LDLR-family receptors with high affinity [86]. Disruption of ApoE lipidation and trafficking changes neuronal membrane composition and can alter the processing of amyloid precursor protein (APP) and amyloid-β (Aβ), as shown by ABCA1-loss models that display low brain ApoE levels and increased amyloid deposition, as well as gain-of-function studies where increasing ABCA1 reduces plaques [87,88]. Beyond Alzheimer-related phenotypes, new work shows that astrocytes can adjust neuronal cholesterol content through ApoE-dependent programs that rewire neuronal physiology [89,90,91,92].

#### 3.3.3. CD36 and Long-Chain Fatty Acid Uptake

CD36 is a high-capacity fatty-acid transporter and scavenger that operates in the neurovascular unit and in parenchymal cells, where it shapes lipid entry, redox stress, and inflammatory signaling [93,94,95,96]. Endothelial and glial CD36 influence how long-chain fatty acids cross neural barriers and feed mitochondrial oxidation, and alterations in CD36 expression can couple vascular function to lipid overload and oxidative injury [95,97]. In peripheral nerves, where myelinating glia face large swings in lipid demand, the level of CD36 becomes a key determinant of whether incoming fatty acids are matched by oxidative capacity or instead accumulate as potentially toxic lipid species [62,98].

#### 3.3.4. ABCA1/ABCG1 and Cholesterol Efflux

ATP-binding cassette transporter A1 (ABCA1) and ATP-binding cassette transporter G1 (ABCG1) mediate cholesterol and phospholipid efflux to apolipoproteins and complete the loop of lipid circulation within the brain, supporting the formation of ApoE-containing particles and protecting cells from sterol overload [87,99]. Loss of ABCA1 lowers brain ApoE, impairs ApoE lipidation, and increases amyloid deposition in mouse models, whereas increasing ABCA1 activity can lessen plaque burden, which illustrates how efflux capacity conditions neuronal lipid balance and proteostasis [88,100]. These transporters are expressed across neural cell types and link intracellular sterol pools to extracellular lipoprotein dynamics [87].

Table 1 and Table 2 summarizes the main receptors and transporters influenced by PCSK9, highlighting their functions, regulatory mechanisms, and neural consequences in CNS and PNS contexts.

### 3.4. Synthesis, β-Oxidation, and Lipid Turnover in Neural Cells

Cholesterol synthesis in neural tissue proceeds through the SREBP2–HMGCR pathway, with astrocytes producing most sterol for export to neurons, while neurons emphasize efficient handling and disposal, including conversion to 24S-hydroxycholesterol for elimination [5,64,101,102]. Fatty-acid synthesis supplies membrane lipids for growth and repair, and peroxisomes extend this capacity by processing very-long-chain species and by generating plasmalogens that stabilize myelin [68]. Mitochondria oxidize long-chain fatty acids via the carnitine shuttle and β-oxidation, and the buildup of acylcarnitines marks a mismatch between lipid entry and oxidative throughput, a pattern reported across insulin-resistant states and relevant to glia under metabolic stress [103,104]. In the PNS, Schwann cells shift between anabolic lipid synthesis for myelin and catabolic oxidation for energy, and mitochondrial insufficiency in Schwann cells triggers maladaptive lipid remodeling that precedes axonal degeneration [10,105]. Together these programs create multiple control points where receptor availability and transporter activity can tip the balance toward healthy turnover or toward lipotoxic stress.

### 3.5. Mitochondrial Lipid Handling and Redox Balance in Myelinating Glia

Myelinating glia require continuous ATP for lipid synthesis, vesicle trafficking and protein folding, so their mitochondria must oxidize fatty acids efficiently without excess reactive oxygen species. When fatty-acid influx exceeds oxidative capacity, acylcarnitines and lipid droplets accumulate and redox buffers are taxed, which activates stress pathways and compromises myelin assembly and maintenance [7,104,106,107]. Work in Schwann cells shows that mitochondrial dysfunction provokes a reprogramming of lipid metabolism that generates toxic lipid species, disrupts axon–glia interactions and leads to neuropathy, highlighting how closely myelin integrity is tied to mitochondrial lipid handling [108,109,110,111]. Peroxisomal support becomes particularly important in this setting, because peroxisomes share the burden of very-long-chain fatty-acid metabolism and contribute precursors for plasmalogens that protect myelin from oxidative damage [11,62,68,112,113].

## 4. PCSK9 in the Central Nervous System

### 4.1. Expression Across Neural Cell Types and Developmental Regulation

PCSK9 was first identified in neurons as neural apoptosis-regulated convertase 1, which already suggested an intrinsic neural role distinct from hepatic cholesterol control, and in the adult brain its expression is generally low yet detectable, with signals enriched in regions that retain plasticity or active remodeling such as neurogenic niches and limbic circuits where synapses and dendrites turn over more rapidly [4,17,114,115,116]. Although PCSK9 is measurable in cerebrospinal fluid at concentrations far below those in serum, and although access from the circulation into brain parenchyma is limited by the endothelial barrier, most functional effects in the central nervous system are therefore expected to arise from protein produced locally by neural cells rather than from the circulating pool [4,16,17].

Across development and in injury paradigms, neural progenitors increase PCSK9 as they adopt a neuronal fate, and differentiating neurons tend to maintain expression in a pattern that does not mirror classic sterol-responsive programs described for the liver, which points to context-specific regulation tied to lineage decisions, membrane growth, and cellular stress [3,15,117,118]. In healthy adult tissue, single-cell and regional profiling converge on a picture of modest baseline expression that can rise in defined disease states, which is consistent with a role that is switched on when plasticity, turnover, or inflammatory pressure become prominent. Astrocytes and microglia express little at rest but both cell types can upregulate PCSK9 when exposed to inflammatory cues, creating a route by which lipid-handling programs intersect with innate immune signaling, while oligodendroglial lineage cells show low basal expression that may increase during active myelin remodeling, so the overall distribution across the brain is best described as sparse at baseline yet inducible in neurons and glia when remodeling or stress demands it [62,119,120,121,122,123].

### 4.2. Neurodevelopment and Synaptic Biology: Reelin Signaling Through ApoER2 and VLDLR

The Reelin pathway organizes neuronal migration during development and continues to shape synaptic structure in adulthood. ApoER2 and VLDLR act as coreceptors for Reelin, and their activation triggers Dab1 and Src family kinases, increases trafficking and the function of N-methyl-D-aspartate (NMDA) receptors at postsynaptic sites, and supports long-term potentiation and stable memory formation. Because PCSK9 can reduce the surface abundance of ApoER2 and VLDLR, it has the capacity to attenuate Reelin signaling and to shift synaptic physiology toward lower plasticity [8,17,124]. In cultured neurons, higher PCSK9 lowers LDL receptor family levels and reduces neurite complexity, whereas silencing PCSK9 preserves receptor availability and maintains outgrowth. In vivo the picture is more nuanced because whole body Pcsk9 knockout mice develop normally and show intact baseline behavior, which implies compensation across development and argues for models that remove PCSK9 in specific neural cell types and at selected time points [3,4,8,15,125]. Early neuron-specific manipulations now point to altered spine morphology in the hippocampus and to subtle cognitive changes, which strengthens the idea that PCSK9 tunes synaptic circuits by controlling receptor routing rather than by acting as a protease [16,126].

### 4.3. Apoptosis, Stress, and Inflammatory Signaling Linked to PCSK9

Work in neurons supports a model in which PCSK9 promotes apoptosis by lowering ApoER2 and related survival pathways [126]. Reducing PCSK9 by RNA interference or by genetic means limits caspase activation, preserves mitochondrial integrity, and improves survival in trophic withdrawal and toxin exposure paradigms [127,128]. Beyond cell autonomous effects. there is evidence that PCSK9 shapes inflammatory tone in the neurovascular unit. In rodent stroke models, pharmacologic or genetic reduction in PCSK9 lowers cytokine release, reduces microglial activation, and improves tissue outcomes, with signaling changes that map to Toll-like receptor and nuclear factor kappa-light-chain-enhancer of activated B cell (NF-κB) pathways in microglia and endothelium [15,129,130]. Across trophic, toxic, and ischemic stress, the direction of effect is consistent, since less PCSK9 associates with lower apoptotic signaling and less inflammation, while the magnitude depends on cell type, timing, and the availability of LDL receptor family targets [4,8].

### 4.4. PCSK9 in Neurodegenerative and Demyelinating Contexts

Human studies in Alzheimer’s disease show mixed results. Some cohorts report higher cerebrospinal fluid PCSK9 and links to apolipoprotein E4 (APOE4), whereas others find no difference between patients and controls, likely reflecting differences in assays, sampling strategies, and disease stage [131,132]. Mechanistically, an indirect route is most plausible because ApoE-containing lipoproteins and the LDL receptor family govern neuronal lipid delivery and amyloid handling, so PCSK9-driven changes in receptor availability could shift ApoE-dependent trafficking even if PCSK9 does not act directly on amyloid [133,134]. Evidence for a role in primary demyelination is limited. In experimental autoimmune encephalomyelitis, altering PCSK9 does not change clinical course or canonical immune readouts, and reviews of multiple sclerosis do not place PCSK9 among core disease mechanisms despite the central importance of lipid metabolism in myelin biology [135]. By contrast, acute cerebrovascular injury shows a more consistent pattern in animals: across rodent stroke models, lowering PCSK9 before or around the ischemic insult reduces infarct size and improves functional recovery, consistent with an anti-inflammatory, tissue-protective effect [136,137,138]. Translation to patients will require trials designed to separate lipid-lowering benefits from any direct actions within the CNS.

### 4.5. Knowledge Gaps and Methodological Considerations: Local vs Circulating PCSK9 and Barrier Constraints

Three factors limit firm conclusions about PCSK9 in the CNS. First, the blood–brain barrier restricts the entry of circulating PCSK9, and cerebrospinal fluid concentrations are much lower than in serum, so plasma measurements do not serve as a proxy for brain biology and studies should prioritize local readouts [139]. In humans, cerebrospinal fluid (CSF) PCSK9 is typically in the low-ng/mL range (≈3–5 ng/mL) and thus far below diurnal serum levels (hundreds of ng/mL), as shown across cohorts and reviews. [4,15,140,141]. Second, many insights come from neuron cultures or global knockouts that allow developmental compensation, therefore inducible and cell-type-specific manipulations in neurons, astrocytes, microglia, and oligodendrocytes are needed to test whether PCSK9 primarily regulates receptor routing, inflammatory tone, or both in mature circuits. Current reviews emphasize the low baseline expression in adult brain, context-dependent upregulation, and the need for temporally controlled, cell-specific models to resolve neuronal versus glial roles [142,143]. Third, human datasets disagree about changes in cerebrospinal fluid PCSK9 across diseases, arguing for standardized pre-analytical handling, clear reporting of antibody epitopes that distinguish full-length from furin-cleaved forms, longitudinal sampling across disease stages, and stratification by APOE genotype. Studies in the AD continuum report both null findings and associations with APOE4 or higher CSF PCSK9, highlighting assay and cohort heterogeneity [144,145,146]. Finally, because ApoER2 and VLDLR serve as both lipid receptors and Reelin receptors, experiments should read out receptor trafficking and circuit-level physiology in parallel so that changes in lipid supply can be distinguished from changes in synaptic signaling; distinguishing full-length from furin-cleaved PCSK9 is methodologically important because these forms differ biochemically and are differentially detected by antibodies [30,147]. 

**Figure 2:**
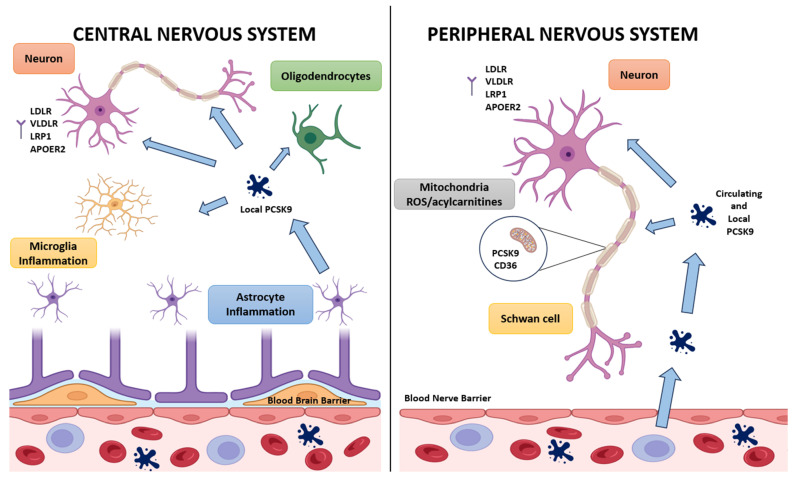
Comparison of PCSK9 roles in the central and peripheral nervous system.

An overview comparing PCSK9 biology in the central and peripheral nervous system is provided in Figure 2, which highlights differences in cellular sources, barrier access, receptor targets, and downstream consequences for lipid handling and glial integrity.

In the CNS, PCSK9 is produced locally by neurons, astrocytes, oligodendrocytes, and microglia, while circulating PCSK9 has negligible entry due to the restrictive blood–brain barrier. Local PCSK9 modulates members of the LDL receptor family (LDLR, VLDLR, LRP1, ApoER2), thereby influencing lipid uptake, receptor routing, neuronal differentiation, and synaptic plasticity. It can also lower pro-survival receptor abundance and promote apoptotic pathways in neurons, while astrocytic and microglial PCSK9 may couple lipid metabolism to neuroinflammation and barrier function. In the PNS, both circulating and locally expressed PCSK9 act on neurons and Schwann cells, as access is less restricted across the blood–nerve barrier. Here, PCSK9 regulates CD36 abundance at the Schwann-cell surface, shaping the balance of long-chain fatty-acid influx. In PCSK9 deficiency, elevated CD36 drives lipid overload, expansion of lipid droplets, acylcarnitine accumulation, and mitochondrial cristae disruption, which together contribute to small-fiber hypomyelination, C-fiber swelling in Remak bundles, and sensory conduction deficits. This defines a PCSK9–CD36–mitochondria axis that links systemic lipid handling with local metabolic vulnerability in peripheral nerves.

## 5. PCSK9 in the Peripheral Nervous System: Focus on Schwann Cells

### 5.1. Expression Across Schwann Cells, Satellite Glia, and Nociceptive Schwann Cells

PCSK9 is present in peripheral nerves at low but detectable levels, with in situ hybridization and immunostaining identifying mRNA and protein in mouse dorsal root ganglia and sciatic nerve, as well as in primary sensory neurons and Schwann cells; human tibial nerve also shows the PCSK9 signal, indicating that the PNS can be a local source in addition to the liver-derived circulating pool [8]. Earlier reviews were cautious, noting that firm evidence for PNS expression was limited to transcript detection in a rat Schwann cell line, which is now complemented by these newer mouse and human data [4,8]. Beyond myelinating and Remak (non-myelinating) Schwann cells, specialized nociceptive Schwann cells in skin form a meshwork around nociceptor endings and participate in mechanical and thermal pain transduction; their functional importance has been confirmed by optogenetic and silencing studies, and their number is reduced in Pcsk9-null mice, linking PCSK9 biology to this glial sensory interface [4,148].

Satellite glial cells in sensory ganglia are abundant, astrocyte-like supporters of neuronal metabolism and signaling, and while PCSK9 expression in this subtype has not been mapped systematically, their lipid-handling reliance on LDLR-family traffic makes them plausible downstream targets of PCSK9-dependent receptor routing [4,8,15,149,150,151,152,153,154].

### 5.2. Genetic PCSK9 Deficiency and Neuropathy Phenotypes

#### 5.2.1. Behavioral and Electrophysiological Readouts

Global Pcsk9^−/−^ mice develop a selective sensory phenotype that appears in early adulthood and becomes more pronounced with age. At 10 weeks males already show reduced responses to light mechanical stimuli and altered thermal pain sensitivity, and by 24 weeks they display blunted responses to both acute mechanical and thermal pain, while dynamic light touch, motor coordination, and grip strength remain intact, which points to small-fiber dysfunction rather than a global motor deficit [8]. Females exhibit a milder or absent phenotype in the same assays; therefore, most downstream analyses focus on males. Sensory nerve conduction velocity is reduced in knockouts whereas motor conduction is preserved, and this physiological pattern co-occurs with normal axon counts, which indicates that early impairment reflects changes in fiber quality and myelin function rather than axon loss. Stimulation and recording were standardized and reported in detail, which supports the specificity of the sensory conduction deficit [8,155].

#### 5.2.2. Hypomyelination and G-Ratio Changes

Morphometry reveals selective thinning of the myelin sheath among small Aδ fibers in Pcsk9^−/−^ nerves. Blinded analysis of more than one thousand axons per genotype shows a significant upward shift in g-ratio that is consistent with hypomyelination, while the distribution of myelinated axon diameters and total axon numbers is unchanged, which identifies myelin thickness rather than axon caliber or abundance as the primary variable affected and explains the slowing of sensory conduction observed physiologically [8,155,156].

#### 5.2.3. Axonal Pathology in Remak Bundles

Unmyelinated C-fiber organization within Remak bundles is disrupted in Pcsk9^−/−^ mice, with transmission electron microscopy showing axonal swelling, abnormal fasciculation, intrabundle vacuoles, and mitochondrial abnormalities, and the fraction of C-fibers with larger-than-typical diameters is increased. In the skin, intraepidermal nerve fiber density is not consistently reduced at later time points, yet the number of specialized nociceptive Schwann cells is lower, which provides a cellular correlate for diminished mechanical pain behaviors and indicates early small-fiber injury centered on Remak units and nociceptive Schwann cells rather than widespread axon loss [8,157,158,159].

In peripheral nerves of Pcsk9^−/−^ mice, CD36 is upregulated in both myelinating and non-myelinating Schwann cells and in axons, whereas other canonical PCSK9 targets such as LDLR, LRP1, VLDLR, and ApoER2 are not detectably altered in this tissue. Nerve lipid content increases, acylcarnitines accumulate, and the mitochondrial ultrastructure is distorted, which together indicates fatty-acid influx that exceeds oxidative capacity. This pattern is congruent with prior work in non-neural tissues showing that PCSK9 promotes CD36 degradation through endo-lysosomal and proteasomal routes, so loss of PCSK9 removes a brake on CD36 and raises lipid entry [4,20,160,161,162].

CD36 also acts as a key scavenger receptor during debris clearance after nerve injury. Mice lacking CD36 remyelinate more slowly after sciatic crush, and pharmacological upregulation of CD36 can accelerate repair, which underscores a dual role in which CD36 is beneficial during acute regeneration yet potentially harmful at baseline if it chronically elevates fatty-acid flux into Schwann cells [10,99,160,161,163,164].

Independent models with primary mitochondrial deficits restricted to Schwann cells reproduce C-fiber swelling, small-fiber dysfunction, and axonopathy without early axon loss. These converging observations support a causal chain from glial metabolic stress to small-fiber injury and position lipid overload as a proximal stressor in Pcsk9-null nerves [10,27,164,165].

### 5.3. Lipid Dysregulation in Peripheral Nerves: Droplets and Raft Composition

In peripheral nerves, lipid imbalance reflects changes in membrane composition and in storage dynamics rather than a single receptor event, because myelin is unusually rich in cholesterol, glycosphingolipids, and plasmalogens that organize raft microdomains, and because these rafts position adhesion molecules and signaling complexes that stabilize paranodes and nodes, so even modest shifts in the ratios of these classes, or the diversion of fatty acids from membrane building toward neutral storage during stress, can loosen axo-glial contacts and reduce the fidelity of impulse propagation [109,116,166].

Schwann cells meet these demands by combining de novo lipogenesis with lipid recycling, and during injury they internalize and digest myelin through myelinophagy and TAM-receptor–dependent phagocytosis, while lipid droplets act as transient buffers that sequester triacylglycerols and cholesteryl esters and hand off cargo to mitochondria and lysosomes as needs change, yet when droplet load rises chronically, it signals that influx is outpacing utilization and it associates with slower conduction and reduced physiological reserve [167,168,169,170].

At the metabolic level, a sustained excess of long-chain fatty acids over mitochondrial capacity yields incomplete β-oxidation, rising acylcarnitines, and cristae disruption in Schwann cells and small fibers, which together mark a mismatch between lipid entry and oxidative handling and predict vulnerability of thin myelin and Remak units; in this context, programs that set fatty-acid entry at the surface, most notably those that determine CD36 abundance, become upstream amplifiers of the membrane and storage phenotypes described here. [11,171,172,173].

### 5.4. Macrophages and Nerve Regeneration: Debris Clearance and CD36

After nerve injury, efficient clearance of myelin debris by macrophages and Schwann cells is a prerequisite for timely remyelination; CD36 on phagocytes is a key scavenger that accelerates myelin uptake and resolution of inflammation, and its loss delays remyelination in the peripheral nerve crush model [174]. In demyelinating contexts, boosting CD36-dependent debris uptake reduces inflammatory burden in vivo, and small-molecule or nutritional interventions that enhance CD36 expression can rejuvenate debris clearance, illustrating how lipid-scavenging capacity directly shapes repair [168,174]. Because PCSK9 can downregulate CD36 in peripheral tissues, decreased PCSK9 activity might raise CD36 availability not only on Schwann cells but also on infiltrating or resident macrophages, with a two-edged consequence: improved debris clearance during regeneration but increased fatty-acid flux and lipid droplet load at baseline; resolving these context-dependent outcomes will require cell-type-specific manipulations of Pcsk9 and CD36 during injury and repair [8,17,175].

### 5.5. Pharmacological Inhibition of PCSK9 in Diabetic Peripheral Neuropathy

Recent pharmacological data complement the genetic findings by showing that PCSK9 inhibition may exert beneficial effects on peripheral nerves under metabolic stress. In a rat model of diabetic peripheral neuropathy induced by a high-fat diet and streptozotocin injection, treatment with the monoclonal antibody Alirocumab improved both functional and structural outcomes [23]. Specifically, Alirocumab enhanced sensory nerve conduction, mitigated small-fiber deficits, and attenuated morphological abnormalities of the sciatic nerve. These benefits were associated with reduced oxidative stress and decreased inflammatory responses in peripheral nerves, suggesting that PCSK9 blockade may confer protection by modulating metabolic and immune pathways in addition to lipid handling. While these results differ from the detrimental phenotype observed in constitutive Pcsk9 deficiency, they highlight that pharmacological inhibition in adulthood, acting primarily in the circulation and liver, may have context-dependent neuroprotective effects in acquired neuropathies such as diabetes. This underscores the need to distinguish carefully between germline Pcsk9 loss and therapeutic inhibition when considering translational implications for peripheral nerve health [23].

## 6. The PCSK9–CD36–Mitochondria Axis: A Working Model

### 6.1. Evidence That PCSK9 Regulates CD36 Abundance and Trafficking

A large body of work outside the nervous system shows that PCSK9 reduces cell-surface CD36 and promotes its degradation, which lowers fatty-acid uptake and limits triglyceride storage [21,58]. In genetic PCSK9 loss, CD36 levels rise in several tissues, and this change is accompanied by higher lipid influx and larger neutral-lipid stores, which indicates that PCSK9 normally acts as a brake on CD36-dependent transport [17,20,21,176,177]. Recent nerve studies are consistent with this view because CD36 increases in the peripheral nerves of PCSK9-deficient mice, and the increase is most evident in myelinating and non-myelinating Schwann cells and in small fibers, while dorsal root ganglia, which express very little CD36, are largely unchanged. The effect appears post-transcriptional because canonical LDL receptor family targets are not consistently altered in nerve tissue, which focuses attention on CD36 routing. Proper CD36 function also requires correct delivery to the plasma membrane, and proteins such as caveolin-1 control this step, so any perturbation that changes the balance between internal pools and the membrane pool will further modify fatty-acid flux even when total CD36 protein is stable. Together, these observations support a mechanism in which PCSK9 sets the ceiling for functional CD36 at the Schwann-cell surface and thereby limits the rate of lipid entry under physiological conditions [4,15,21,46].

### 6.2. Consequences for Fatty-Acid Influx in Schwann Cells

Schwann cells build large amounts of membrane and therefore need steady supplies of fatty acids and cholesterol, which they obtain by combining de novo synthesis with receptor-mediated uptake [69,178,179,180]. When CD36 abundance rises, the rate of long-chain fatty-acid entry increases, and the incoming lipids are partitioned between membrane synthesis, storage in lipid droplets, and mitochondrial β-oxidation. If influx outruns the need for new membrane and outstrips the capacity of β-oxidation, neutral-lipid droplets expand and signaling lipids such as diacylglycerols and ceramides accumulate, which can disturb kinase signaling and endoplasmic-reticulum proteostasis [98,181,182,183,184]. At the same time, receptor-mediated uptake competes with cholesterol efflux pathways driven by ABCA1 and ABCG1, so an increase in import without a matched increase in export can shift the composition of raft-forming lipids in myelin. Because membrane order and protein packing in myelin depend on cholesterol and glycosphingolipid balance, any chronic skew in these pools will make myelin thinner and less stable even when axon caliber and axon number remain normal. In this setting, Schwann cells begin to rely more heavily on mitochondrial oxidation to dispose of excess fatty acids, which sets the stage for organelle stress [10,21,98,164].

### 6.3. Mitochondrial Overload, Reactive Oxygen Species, and Bioenergetic Failure

Mitochondria import long-chain acyl groups through the carnitine shuttle that is controlled by CPT1 at the outer membrane, by the carnitine–acylcarnitine carrier in the inner membrane, and by CPT2 at the matrix side, and the imported substrates are then processed by β-oxidation and fed into the tricarboxylic-acid cycle and the electron-transport chain [10]. When substrate delivery is persistently high relative to enzymatic capacity, electrons accumulate at respiratory complexes and leak to oxygen, which increases reactive oxygen species and oxidizes lipids and proteins within the organelle [185]. Cardiolipin at the inner membrane is particularly sensitive to peroxidation, and its damage destabilizes respiratory supercomplexes and promotes cytochrome-c release, which amplifies cell-stress signaling [186]. Schwann cells cope for a time by activating the integrated stress response and by increasing mitophagy, yet prolonged mismatch lowers ATP output and reduces the pool of high-energy phosphates that is needed for lipid synthesis, protein folding, and vesicular traffic [187,188]. Independent models in which Schwann-cell mitochondria are genetically impaired reproduce small-fiber dysfunction and demyelination, which indicates that mitochondrial health by itself is a critical determinant of peripheral-nerve integrity [164]. The PCSK9–CD36 axis plugs directly into this vulnerability because it controls the amount of lipid substrate that reaches the organelle.

### 6.4. Acylcarnitine Buildup as a Signature of Incomplete β-Oxidation

Acylcarnitines form when long-chain acyl-CoAs are transferred to carnitine and shuttled across the inner membrane, and they normally turn over quickly as β-oxidation proceeds. When oxidation stalls at any step, acylcarnitines accumulate and can be measured as a fingerprint of incomplete oxidation. In peripheral nerves from PCSK9-deficient mice, acylcarnitines rise together with CD36 abundance and with visible mitochondrial distortion in small fibers and in Schwann cells, which links the biochemical signature to both the transport change and the organelle phenotype in the same tissue [17,43,129,171]. The chain-length pattern is informative because elevations of medium- and long-chain species point to a matrix bottleneck, whereas very-long-chain species also reflect peroxisomal load. Beyond being markers, acylcarnitines themselves can stress membranes and disturb mitochondrial potential, which creates a feed-forward loop in which incomplete oxidation begets more incomplete oxidation. In nerves, this translates into reduced energy available for myelin maintenance and into higher oxidative burden in Remak units that are already metabolically fragile [8,164,189,190,191].

### 6.5. Integrated Model for Hypomyelination and Small-Fiber Injury

We propose that PCSK9 acts in peripheral nerves to keep CD36-dependent lipid entry aligned with the anabolic and oxidative capacity of Schwann cells. When PCSK9 activity is intact, CD36 at the Schwann-cell surface remains in a range that supports membrane synthesis without forcing excess substrate into mitochondria [4,46], lipid droplets remain modest in size, and β-oxidation products do not accumulate. When PCSK9 is absent, CD36 increases on myelinating and non-myelinating Schwann cells and on adjacent fibers, fatty-acid influx rises above demand, lipid droplets expand, and acylcarnitines build up, while mitochondria show cristae disruption and a lower ability to sustain ATP production during continuous myelin upkeep. The energetic shortfall and the oxidative burden together reduce the thickness and quality of small-fiber myelin and shift the g-ratio upward, while unmyelinated C-fibers develop axonal swellings and disorganized Remak architecture [192]. Functionally, sensory conduction slows and mechanical and thermal pain sensitivity decline, which matches the selective small-fiber phenotype reported in genetic PCSK9 loss. In injury settings the axis becomes context-dependent because CD36 on macrophages is required for efficient myelin debris clearance and for timely remyelination, so higher CD36 may aid regeneration after damage even while it risks lipotoxic stress in the uninjured state. The model therefore predicts that Schwann-cell-specific manipulation of PCSK9 or CD36 will dominantly control baseline myelin and small-fiber integrity, whereas myeloid-specific manipulation will dominantly control the tempo of debris clearance and repair. It also predicts that partial reduction in CD36 in Schwann cells, or pharmacological tempering of fatty-acid entry, will normalize acylcarnitines and the mitochondrial ultrastructure and will restore conduction without changing axon counts, while broad inhibition of mitochondrial import such as high-dose CPT1 blockade would be harmful because it would further trap acyl groups upstream and worsen lipid buildup [8,193,194]. Direct tests could combine conditional genetics with isotope tracing of ^13^C-palmitate in ex vivo nerves to quantify flux into acylcarnitines and carbon entry into the tricarboxylic-acid cycle, together with high-resolution respirometry and blinded g-ratio morphometry, which would allow this pathway to be mapped quantitatively from transporter to organelle to structure to function (Table 3).

This table summarizes how PCSK9 deficiency alters CD36 abundance and lipid influx in Schwann cells, leading to mitochondrial overload, acylcarnitine accumulation, and structural changes in myelin and axons. The cascade illustrates a mechanistic link from receptor regulation to small-fiber dysfunction and neuropathy.

## 7. Clinical Implications: PCSK9 Inhibitors and Neural Safety

### 7.1. Pharmacologic Inhibition Versus Genetic Loss: What Differs and Why It Matters

Genetic loss of PCSK9 removes the protein from conception in every tissue, which allows developmental compensation and exposes peripheral nerves to a lifelong shift in receptor routing, fatty-acid handling, and mitochondrial workload, whereas pharmacologic inhibitors act in adulthood, reduce but do not abolish PCSK9 activity, and work primarily in the circulation and liver [195]. Monoclonal antibodies such as evolocumab and alirocumab bind extracellular PCSK9 and prevent it from targeting LDLR for degradation, while inclisiran, a GalNAc-conjugated siRNA, lowers hepatic PCSK9 synthesis, neither approach is designed to penetrate the brain, and large IgG antibodies in particular have negligible access across the blood–brain barrier under physiological conditions [76,196]. The blood–nerve barrier is more permissive than the blood–brain barrier to small solutes, yet it still restricts immunoglobulins, which means systemic PCSK9 antibodies are unlikely to reach endoneurial compartments at meaningful levels, so their neural effects are expected to be indirect through altered lipoprotein flux or immune tone rather than direct receptor editing in situ. These pharmacologic features differ sharply from full Pcsk9 knockout in mice, where peripheral nerves show increased CD36, excess lipid entry, and mitochondrial stress, so translation requires caution because dosage, tissue exposure, and the timing of inhibition are fundamentally different between drugs and germline models [8,43,75,197,198,199,200].

Moreover, it is important to emphasize that currently approved PCSK9 inhibitors, such as evolocumab and alirocumab, are designed to block the interaction of circulating PCSK9 with LDLR, thereby preventing receptor degradation and lowering plasma LDL cholesterol. These antibodies do not interfere with other PCSK9 targets described in experimental models, including CD36, VLDLR, or ApoER2, nor do they mimic the global absence of PCSK9 from conception. As a result, the metabolic and neural consequences of therapeutic inhibition are fundamentally narrower in scope than those observed in full Pcsk9 knockout mice, where derepression of CD36 contributes to lipid overload and small-fiber stress [8,43,75,197,198,199,200]. Distinguishing between target selectivity in pharmacological blockade and the pleiotropic effects of genetic loss is therefore critical for interpreting preclinical–clinical translation.

### 7.2. Neural Outcomes in Large Cardiovascular Trials: Signals and Limits of Detection

Across randomized outcome programs, blinded cognitive testing and adverse-event surveillance have not shown harm. In the EBBINGHAUS substudy of FOURIER, patients on evolocumab had no decrement in working memory, executive function, or psychomotor speed compared with the placebo, and extended follow-up in EBBINGHAUS-OLE remained neutral, which argues against a clinically important CNS signal during multi-year exposure. ODYSSEY OUTCOMES did not identify an excess of neurocognitive events with alirocumab, and pooled trial reviews and meta-analyses consistently report neutral neurocognitive safety profiles for PCSK9 inhibitors, although these datasets were not powered for small-fiber neuropathy or subtle sensory changes. Inclisiran’s phase 3 ORION program similarly reports no excess neurocognitive adverse events, which aligns with its liver-targeted design. Together, these data are reassuring for brain outcomes, yet they leave open questions about peripheral small-fiber biology because nerve conduction studies and pain phenotyping were not prespecified primary endpoints in these cardiovascular trials [192,201,202,203,204,205,206,207].

### 7.3. Case Reports and Post-Marketing Pharmacovigilance: How to Interpret

Post-marketing case literature includes rare descriptions of peripheral neuropathy temporally associated with alirocumab, and pharmacovigilance analyses have noted disproportionality signals for some neuropsychiatric terms with PCSK9 inhibitors; however, spontaneous reports lack denominators, have channeling bias, and cannot adjudicate causality, while confounding by comorbidity, concomitant statins, diabetes, or B-12 status is common [3,15,208]. In contrast, adjudicated signals have not emerged from randomized trials or their open-label extensions, which reduces the prior probability that PCSK9 inhibition causes clinically meaningful neuropathy in the general cardiovascular population, though very infrequent idiosyncratic events cannot be excluded. For clinical decision-making, the balance of evidence supports neural safety, with the prudent caveat that new sensory symptoms after initiation should prompt routine evaluation for common causes and not be reflexively attributed to the drug in the absence of corroborating findings [4,209].

### 7.4. Translational Angles: Targeting Lipid Flux Without Harming Myelin

Mechanistic work in Schwann cells shows that myelination depends on steady cholesterol supply, controlled long-chain fatty-acid entry, and tight mitochondrial redox balance, and it also shows that excess fatty-acid influx can produce acylcarnitine buildup and oxidative stress that erodes small-fiber integrity [4,8,141,208,209,210]. These principles suggest several translational guardrails. First, if systemic PCSK9 blockade is combined with other lipid-active therapies, the net effect on peripheral fatty-acid handling should be considered, since CD36 activity in nerves and macrophages influences both lipid overload and debris clearance during regeneration; in mice, augmenting CD36-positive macrophage function can enhance remyelination after injury, whereas unchecked CD36-mediated lipid entry into glia risks mitochondrial overload. Second, PPARγ agonists and LXR agonists can improve nerve phenotypes in metabolic models by shifting transcriptional programs toward lipid efflux and anti-inflammatory states, yet LXR activation can raise triglycerides and drive hepatic lipogenesis, so any attempt to couple PCSK9 inhibition with PPAR/LXR modulation should be staged, monitored, and ideally restricted to trials with predefined neural endpoints [57,211,212,213,214]. Third, early studies indicate that anti-PCSK9 antibodies can lower surface CD36 on some peripheral cells, which raises the possibility that systemic therapy might actually reduce certain CD36-dependent lipid influx pathways, though organ specificity and dosing windows matter and must be resolved directly in human nerve tissue. Finally, omega-3 polyunsaturated fatty acids and lifestyle measures that lower triglyceride-rich lipoprotein exposure may offer a low-risk adjunct to stabilize neural lipid traffic during aggressive LDL-lowering regimens [215,216].

### 7.5. Practical Guidance for Future RCTs in Lipid-Lowering and Neural Safety

Future randomized studies that aim to close the neural safety gap should embed peripheral nerve outcomes from the outset. Small-fiber end points deserve priority because animal and human metabolic neuropathies begin in unmyelinated and thinly myelinated axons. Skin biopsy with intraepidermal nerve fiber density (IENFD) and standardized quantitative sensory testing can detect early loss and altered thresholds, while corneal confocal microscopy offers a rapid, non-invasive readout of small-fiber structure with growing validation for both diagnosis and regeneration; these measures should be paired with symptom inventories and objective autonomic testing [217,218,219]. Nerve conduction studies and late responses remain important for large-fiber surveillance, yet they will miss early small-fiber change, so a tiered battery that combines IENFD, QST, and corneal confocal microscopy with targeted electrophysiology is recommended. Imaging biomarkers such as magnetic-resonance neurography–derived intraneural lipid-equivalent lesions may complement histology by tracking lipid storage stress over time. Trials should stratify by diabetes, obesity, and baseline triglycerides, record concomitant statins and fibrates, and prespecify thresholds for clinically meaningful change, while CNS-focused substudies can reuse the EBBINGHAUS cognitive platform to maintain continuity with prior safety work [207,220,221,222].

### 7.6. PCSK9, Systemic Metabolic Disorders, and Peripheral Neuropathy

Systemic metabolic disorders such as diabetes, obesity, and dyslipidemia represent leading risk factors for peripheral neuropathy, and they share the pathophysiological features of lipid imbalance, oxidative stress, and chronic inflammation. PCSK9 intersects with these processes at several levels. By regulating LDLR-family receptors, PCSK9 shapes circulating lipoproteins that contribute to dyslipidemic risk; by modulating CD36 abundance, it influences fatty-acid entry into Schwann cells and macrophages; and by tuning inflammatory tone, it may amplify or attenuate injury responses in metabolic tissues. In diabetic rodent models, pharmacologic inhibition with alirocumab improved sciatic nerve conduction and reduced oxidative and inflammatory injury [23], suggesting potential therapeutic benefit in the context of metabolic stress. By contrast, germline Pcsk9 deficiency produces chronic CD36 derepression and small-fiber pathology, underscoring that therapeutic inhibition and genetic loss are not equivalent. Together, these observations identify PCSK9 as a mechanistic link between systemic metabolic state and peripheral nerve integrity, and they highlight the need for targeted studies of PCSK9 in neuropathies associated with diabetes, obesity, and dyslipidemia.

## 8. Future Directions and Open Questions

### 8.1. Cell Type Specific Roles Beyond Schwann Cells: Satellite and Enteric Glia

Work to date has centered on Schwann cells, yet peripheral nerves contain several glial populations that may use PCSK9-dependent receptor routing in distinct ways; therefore, the next phase should map PCSK9 expression and function in the satellite glia of dorsal root ganglia, in nociceptive Schwann cells in the skin, and in enteric glia that support visceral sensory circuits, using single-cell and nuclei-resolved approaches that can separate rare subtypes, followed by inducible, cell-type-specific genetics to test how loss or gain of PCSK9 alters receptor abundance, lipid handling, and survival pathways in each population across development and adulthood. Because circulating PCSK9 is restricted by barriers while local production varies with state, studies should compare local deletion with selective reduction in the circulating pool, and should measure full-length and cleaved forms, so that we can decide which compartments are most relevant for each glial subtype and which axis is most tractable for therapy [223,224,225].

### 8.2. PCSK9 in Nerve Regeneration and Pain Biology

After injury, Schwann cells switch into a repair state and macrophages clear myelin debris, while nociceptive Schwann cells shape stimulus detection, therefore experiments that time PCSK9 or CD36 manipulation to the degeneration phase, the debris-clearance phase and the remyelination phase are needed, with parallel readouts of debris load, axon regrowth, remyelination quality, and pain behavior, so that we can separate beneficial effects on clearance from the harmful effects of chronic lipid influx at baseline. Because small fibers drive early sensory symptoms, models should include assays of mechanical and thermal thresholds, spontaneous pain behavior, and conditioned pain modulation, together with sensory nerve conduction and gentle skin biopsy metrics, which will reveal whether modulating the PCSK9–CD36 axis can accelerate recovery without blunting protective sensation or provoking allodynia [21,226,227].

### 8.3. Human Tissue and iPSC-Derived Schwann Cell Models, Spatial and Transcriptomic Mapping

Translation will require human systems that capture lipid flux in glia; therefore, we propose side-by-side studies in iPSC-derived Schwann cells, in neuron–Schwann co-cultures and in human nerve biopsies when available, with standardized culture lipids and defined albumin carriers to avoid confounding. Spatial transcriptomics and proteomics on the human skin and nerve should map PCSK9, CD36, and LDLR-family targets across myelinating and Remak domains, while single-cell datasets should be aligned with targeted lipidomics so that expression changes can be tied to measurable shifts in fatty-acid transport and cholesterol handling. Isotope-tracing with ^13^C-fatty acids, high-resolution respirometry, and quantitative electron microscopy for mitochondrial structure will provide functional anchors, and perturbation screens that silence or overexpress PCSK9 and CD36 will identify rescue points that are conserved between mouse and human glia [17,43,184,228,229].

### 8.4. Biomarkers for Clinical Studies: Lipid Signatures, Mitochondrial Readouts, Imaging

A practical biomarker set should report on the same biology described in nerves, therefore plasma, and, when feasible, microdialysate or tissue extracts should undergo targeted acylcarnitine profiling as a readout of incomplete β-oxidation, while peripheral blood cells can provide complementary mitochondrial stress markers that change with systemic lipid flux [107,230,231]. For structure, magnetic resonance neurography with lipid-equivalent lesion quantification can track the intraneural lipid burden over time, and corneal confocal microscopy can report small-fiber morphology non-invasively, while skin biopsy with intraepidermal nerve fiber density and nodal architecture gives histologic confirmation when needed. Combining these measures with quantitative sensory testing and selected nerve conduction indices will create a multimodal panel that is sensitive to early small-fiber change and that can be repeated during dose finding and follow-up [155,161,226,232,233,234].

### 8.5. Therapeutic Strategies: Schwann-Cell-Specific Modulation and CD36 Fine-Tuning

Because the axis is likely beneficial during debris clearance yet risky during steady-state maintenance, interventions should favor Schwann-cell specificity and partial modulation, not global and permanent blockade. In preclinical work, Schwann-cell-restricted reduction in CD36 or tempering of fatty-acid import should be tested for the ability to normalize acylcarnitine levels, restore mitochondrial structure and improve conduction without reducing axon number, while myeloid-restricted enhancement of CD36 can be timed to injury to accelerate debris removal and remyelination, and the two approaches can be combined with careful scheduling so that repair is helped and baseline lipotoxicity is avoided [156,235,236]. Pharmacology can explore low-intensity CD36 antagonists, PPAR or LXR programs that are titrated to favor efflux and anti-inflammatory tone, and nutritional strategies that lower triglyceride exposure, with prespecified neural endpoints so that benefits on lipid profiles are not pursued at the expense of myelin quality. Finally, if PCSK9 is targeted directly, designs should account for the difference between circulating and local pools and should include exposure measurements in peripheral nerve compartments, so that dosing can be tuned to achieve cardiovascular benefit while maintaining safe lipid flux in Schwann cells [4,8,17,21,139,177].

## 9. Conclusions

PCSK9 regulates the neural lipid economy by tuning the surface availability of receptors that govern cholesterol and fatty-acid entry, and by doing so it influences raft composition, lipid droplet handling, and mitochondrial workload in neurons and glia. In practice this means that PCSK9 helps match lipid supply to the combined demands of membrane building and energy production, which keeps myelin stable and synapses functional.

Its roles differ across the nervous system. In the CNS, low local expression can modulate ApoER2 and VLDLR and can influence stress responses, while in the PNS the dominant story centers on Schwann cells. When PCSK9 is reduced, CD36 rises, fatty-acid influx increases, acylcarnitines accumulate, mitochondria are stressed, and small fibers show thinner myelin and disordered Remak bundles, placing Schwann cells at the heart of peripheral vulnerability.

Clinically, large trials of PCSK9 inhibitors are broadly reassuring for brain safety, yet they were not built to detect small-fiber change, so neuropathy questions need targeted endpoints. A practical path forward is to monitor the biomarkers of incomplete β-oxidation and small-fiber structure and function, and to test therapies that temper Schwann-cell lipid influx while preserving debris clearance after injury. With cell-type-specific models and human iPSC systems, this axis can be translated into focused strategies for preventing or treating lipid-driven neuropathies.

## Figures and Tables

**Table 1:** Key receptors and transporters regulated by PCSK9.

Receptor/Transporter	Primary Functions	Tissue Distribution	PCSK9 Regulatory Effect	Mechanism of Action	Neural Consequences
LDLR Family					
LDLR	• LDL cholesterol uptake • Hepatic lipoprotein clearance • Cellular cholesterol homeostasis	Liver (high), CNS neurons, Peripheral tissues	Promotes degradation, Reduces surface availability	• Binds EGF-A repeat • Prevents receptor recycling • Diverts to lysosomal degradation • Both extracellular and intracellular pathways	• Reduced cholesterol uptake • Altered membrane composition
VLDLR	• VLDL and apoE lipoprotein uptake • Reelin signaling coreceptor • Neuronal migration guidance • Synaptic plasticity	CNS neurons, Astrocytes, Developing brain regions	Reduces surface abundance, Attenuates Reelin signaling	• Similar to LDLR routing • Lysosomal targeting after endocytosis	• Impaired synaptic plasticity • Reduced neurite complexity • Altered spine morphology
ApoER2	• Lipoprotein uptake • Reelin signaling coreceptor • NMDA receptor trafficking • Long-term potentiation • Memory formation	CNS neurons, Hippocampus, Cortical regions	Lowers receptor levels, Reduces Reelin responses	• PCSK9 binding promotes degradation • Interference with recycling adaptors	• Decreased synaptic strength • Cognitive alterations • Reduced neuronal survival
LRP1	• Multifunctional endocytic receptor • Lipid handling and signaling • Amyloid-β trafficking • Proteostasis regulation	CNS (widespread), Vascular cells, Neurons and glia	Promotes receptor degradation	• Biases toward lysosomal delivery • Reduces recycling efficiency	• Altered amyloid handling • Impaired proteostasis • Modified vascular function
Scavenger Receptors					
CD36	• Long-chain fatty acid transporter • Lipid scavenging • Myelin debris clearance • Oxidized LDL uptake	Schwann cells (high), Macrophages, Endothelial cells, Glial cells	Promotes degradation, Reduces fatty acid influx, (*Primary PNS target*)	• Lysosomal and proteasomal pathways • Post-transcriptional regulation • Surface trafficking control	• Loss of PCSK9 → CD36 upregulation • Excessive fatty acid entry • Lipid droplet accumulation • Mitochondrial overload
Cholesterol Efflux					
ABCA1	• Cholesterol efflux to apoA-I • ApoE lipidation in brain • Lipoprotein particle formation • Protection from sterol overload	Astrocytes, Microglia, Peripheral macrophages, Hepatocytes	Indirect regulation, (Not direct PCSK9 target)	• PCSK9 affects upstream cholesterol balance • Influences sterol-responsive transcription	• Enhanced brain ApoE levels • Improved neuronal lipid supply • Reduced amyloid deposition
ABCG1	• Cholesterol efflux to HDL • Complements ABCA1 function • Cellular sterol homeostasis	Neural cells, Macrophages, Peripheral tissues	Indirect effects, (Secondary to receptor changes)	• Responds to altered intracellular sterol pools • Links to PCSK9-LDLR axis	• Modified cholesterol export • Altered membrane composition

**Table 2:** Summary of PCSK9 Effects.

Receptor Class	Normal PCSK9 Function	PCSK9 Deficiency Result	Clinical Relevance
LDLR Family	Increases receptor degradation → Reduces cholesterol uptake	Enhanced receptor availability → Improved cholesterol delivery	• Cardiovascular protection • Potential cognitive benefits
CD36	Decreases surface availability → Limits fatty acid influx	Increased surface expression → Excessive lipid entry	• Peripheral neuropathy risk • Small-fiber dysfunction • Mitochondrial stress
Efflux Transporters	Indirectly modulates via cholesterol balance	Enhanced efflux capacity → Better lipid homeostasis	• Improved neural lipid circulation • Reduced lipotoxicity

Abbreviations: LDLR, low-density lipoprotein receptor; VLDLR, very low-density lipoprotein receptor; ApoER2, apolipoprotein E receptor 2; LRP1, LDLR-related protein 1; CD36, cluster of differentiation 36; ABCA1/ABCG1, ATP-binding cassette transporters A1/G1; CNS, central nervous system; PNS, peripheral nervous system; EGF-A, epidermal growth factor-like domain A.

**Table 3:** The PCSK9–CD36–mitochondria axis in peripheral nerves.

Component	Normal Function	PCSK9 Deficiency Effect	Consequence
PCSK9	Promotes CD36 degradation; limits fatty acid influx	Absent regulation	Loss of brake on lipid entry
CD36	Fatty acid transporter; controlled surface expression	Increased abundance and activity	Enhanced fatty acid uptake
Fatty Acid Influx	Matched to oxidative capacity	Exceeds mitochondrial capacity	Substrate overload
Mitochondria	Efficient β-oxidation; stable cristae structure	Overloaded; structural disruption	Incomplete oxidation; ROS production
Acylcarnitines	Low levels; rapid turnover	Accumulation of multiple chain lengths	Metabolic stress marker
Myelin	Normal thickness; stable composition	Thinning; altered lipid ratios	Small-fiber hypomyelination
Axonal Function	Normal conduction; intact sensation	Slowed conduction; reduced sensation	Clinical neuropathy phenotype

## Data Availability

No new data were created or analyzed in this study. Data sharing is not applicable to this article.
